# Environmental factors associated with human papillomavirus vaccine coverage in adolescents: 2016-2020 analysis

**DOI:** 10.1590/1518-8345.6285.3804

**Published:** 2022-11-25

**Authors:** Bianca Maria Oliveira Luvisaro, Thales Philipe Rodrigues da Silva, Tercia Moreira Ribeiro da Silva, Sheila Aparecida Ferreira Lachtim, Janaina Fonseca Almeida Souza, Fernanda Penido Matozinhos

**Affiliations:** 1 Universidade Federal de Minas Gerais, Escola de Enfermagem, Belo Horizonte, MG, Brazil

**Keywords:** Immunization, Vaccination Coverage, Adolescent, Papillomaviridae, Epidemiology, Regression Analysis

## Abstract

**Objective:**

to analyze the association between the factors of the social environment and the coverage rates of the human papillomavirus (HPV) vaccine in adolescents, in the State of Minas Gerais, Brazil.

**Method:**

this is an epidemiological, ecological study, with panel and trend analysis from 2016 to 2020. The population consisted of adolescents aged 9 to 13 years. The environmental variables were coverage rates, the municipal index of human development, income, education, and longevity; and the rate of violence. The Prais-Winsten autoregression and the panel regression model were used, in addition to the estimate of the mean annual percentage variation.

**Results:**

the vaccination coverage rates are below the goals recommended by the Ministry of Health for all regions analyzed. Moreover, these rates are associated with factors related to the application of the first dose and to aspects inherent to the social environment, such as the rate of violence.

**Conclusion:**

our findings showed that, although tenuous, aspects of the environment, in addition to individual characteristics, provide relevant information to understand the occurrence of health outcomes, since this vaccination campaign presents a strong influence of the environment and age as factors associated with the low rates.

## Introduction

The human papillomavirus (HPV) is one of the most prevalent sexually transmitted infections in the world^1^. HPV is a virus capable of infecting the skin and mucous membranes of humans and, depending on its subtype and the persistence of the infection, is associated with the appearance of anogenital and cutaneous warts and neoplasms of the anogenital tract, the most frequent being cervical cancer^1^ ^-^ ^2^.

Cervical cancer is a preventable, curable disease with high morbidity and mortality worldwide. In Brazil, this type of cancer ranks third among malignant neoplasms in women and fourth in mortality^2^. The main strategy to control this neoplasm is primary prevention using the HPV vaccine^1^.

The HPV vaccine was introduced worldwide more than ten years ago, but many countries still find barriers to maintain vaccination coverage rates at recommended levels^1^ ^,^ ^3^. In Brazil, the goal estimated by the Ministry of Health (MS), for each dose, is 80%^4^ ^-^ ^5^. Previous studies prove that the goal recommended by the MS covers a large part of the target population, adolescents, since this vaccine should preferably be administered before the beginning of sexual life due to the natural history of the disease. Moreover, this goal aims to achieve collective protection against HPV, thus avoiding cervical cancer^1^ ^,^ ^3^ ^-^ ^6^.

In 2020, the World Health Organization (WHO) launched a global strategy for the elimination of cancer, establishing the increase of goals by the year 2030^3^. One of these goals is for countries to reach a 90% HPV vaccination rate for all target audiences, i.e., girls and boys aged 9 to 15 years^3^.

In Brazil, the implementation of the HPV vaccine in the calendar of the National Immunization Program (PNI) occurred in 2014 and, since then, a vaccination routine has been gradually established^4^ ^-^ ^5^. Currently, this vaccine is offered to both female and male adolescents, in the age groups 9 to 13 years and 11 to 14 years, respectively, in a two-dose regiment, with a six-month interval in between^4^. To achieve fully effective immunity, the vaccine must be applied in its complete regimen, that is, in two doses, according to the age group and the established criteria^4^ ^-^ ^5^.

Regarding HPV vaccine coverage rates in Brazil, they are not considerably different from vaccine coverage in other countries but are insufficient to achieve the global goal for the eradication of cervical cancer^3^ ^,^ ^6^ ^-^ ^7^. In 2014, soon after its implementation, 87% of Brazilian municipalities reached the goal recommended with the first dose, but only 32% of them reached the goal recommended with the second dose^6^. Currently, the coverage rates of the second dose are increasingly lower throughout the Brazilian territory.

The analyses on the vaccination strategy of the first and second dose of this vaccine show that the first was performed on a large scale and with great coverage by the media, being widely disseminated and offered in municipal and state schools^6^ ^-^ ^8^. This aspect may justify the above average rates recommended in almost the entire national territory. Regarding the second dose and subsequent years, however, the HPV vaccine started to be offered only in health centers, which may be one of the reasons for the low rates of vaccination coverage^6^ ^-^ ^8^.

The search for increased vaccination rates for adolescents creates many challenges. Among which, we highlight the decision to vaccinate, either made by those responsible for the adolescents or by the adolescents’ themselves. Regarding the rejection of this vaccine, it occurs mostly due to the spread of fake news^8^.

Overall, the acceptability of vaccines is a complex process that can be affected by several factors. Regarding the decision to vaccinate, in 2014, the Strategic Advisory Group of Experts (SAGE) sought to understand the determinant factors of vaccination^9^.

The group developed a model organized in three main areas: (1) contextual influences: historical, sociocultural, environmental, health system/institutional, economic or political factors; (2) individual and group influences: arising from the personal perception of the vaccine or influences of the social environment; and (3) specific vaccines issues: which are those directly related to the characteristics of the vaccine or the process of vaccination^9^. The term “vaccination hesitation” was defined as the delay in accepting or the rejection to the vaccine, despite its availability^9^.

Other contributing factors are added to the adolescents’ low access to vaccines, such as: social and economic vulnerability and issues related to the difficulties for this public to access health services, often because they do not seek care and, when they seek it, they find little guidance (few of which are correct) from health professionals^1^ ^,^ ^8^ ^,^ ^10^ ^-^ ^12^. Thus, we can observe the significant role the nursing professional plays in this scenario. In Brazil, this professional is mostly responsible for the management of primary health care services and is at the forefront of vaccination campaigns, playing an extremely politically relevant role in gaining a better adhesion of adolescents, besides being a reference for the health team^7^ ^-^ ^8^ ^,^ ^10^ ^-^ ^12^.

Given this context and a lack of Brazilian literature on vaccination and environment specific to the adolescent public, we seek to understand the factors related to the low vaccination rate of HPV vaccination among adolescents, especially from the perspective of the social environment. From the point of view of individual factors, the scientific literature shows that low educational level, low income, difficulty in accessing health services, place of residence, dogmas brought by the media and religious beliefs, in addition to low knowledge and information about the epidemiology of HPV cancer and its vaccine, are predictors for low vaccination rate within this public^6^ ^-^ ^7^ ^,^ ^9^ ^-^ ^13^.

Thus, this study aims to analyze the association between the factors of the social environment and the coverage rates of the human papillomavirus vaccine in adolescents from the State of Minas Gerais (MG), Brazil.

## Method

### Study design

This is an epidemiological, ecological study, in which panel and trend analysis were carried out.

### Location

The study was conducted in the state of Minas Gerais, Brazil.

### Period

The study was conducted from 2016 to 2020.

### Study population

The study population consisted of female and male adolescents, aged 9 to 13 years, who were vaccinated against HPV.

The period and age group framing of this study is justified since it focuses the target audience of the vaccine in question and encompasses the completeness/consolidation of the online system data from 2016.

The state of Minas Gerais consists of 853 municipalities, divided into 19 Regional Health Superintendence (*Superintendências Regionais de Saúde* - SRS) and 9 Regional Health Management Units (*Gerências Regionais de Saúde* - GRS). The municipalities are delimited from cultural, economic, and social identities and shared transport networks and networks, with the purpose of integrating the organization and planning of health actions and services^14^.

They are Alfenas, Barbacena, Belo Horizonte, Coronel Fabriciano, Diamantina, Divinópolis, Governador Valadares, Itabira, Ituiutaba, Januária, Juiz de Fora, Leopoldina, Manhuaçu, Montes Claros, Passos, Patos de Minas, Pedra Azul, Pirapora, Ponte Nova, Pouso Alegre, São João Del Rei, Sete Lagoas, Teófilo Otoni, Ubá, Uberaba, Uberlândia, Unaí, and Varginha.

### Study variables 

All data from this study were extracted through secondary databases, available on government websites.

The HPV vaccine data were extracted from the Information System of the National Immunization Program (SI-PNI), available in the public domain (http://sipni.datasus.gov.br/si-pni-web/faces/inicio.jsf).

Demographic data were extracted from the platform of the Atlas of Human Development in Brazil (http://atlasbrasil.org.br/consulta/planilha), and data on social characteristics were obtained by the State Department of Justice and Public Security (*Secretaria de Estado de Justiça e Segurança Pública* - SEJUSP) (http://www.seguranca.mg.gov.br/2018-08-22-13-39-06/dados-abertos).

In this study, the variable of interest (dependent) was the coverage rate of the second dose (D2) of the HPV vaccine, due to studies related to its immunogenicity, efficacy, and effectiveness^4^ ^-^ ^5^. Thus, individuals must have taken the first dose (D1) and, subsequently, D2.

The independent variables were selected through a literature review. Those that demonstrated a possible association with the variable of interest were considered^1^ ^,^ ^6^ ^-^ ^8^ ^,^ ^10^ ^-^ ^13^. Among which, the following were mentioned: the coverage rates of HPV vaccine D1; municipal human development index (MHDI); municipal human development index income (MHDI income); municipal human development index education (MHDI education); municipal human development index longevity (MHDI longevity), and violence rate *per* 100,000 for each SRS/GRS.

The rate of violence comprises consolidated data on violent crimes: sum of the records of consummated rape; consummated rape of vulnerable; attempted rape of vulnerable; attempted rape; consummated extortion; attempted extortion; extortion by consummate kidnapping; attempted homicide; consummated theft; attempted theft; consummate kidnapping and false imprisonment; attempted kidnapping and false imprisonment; and consummated homicide. For the consistency of the study, pre-analysis tests were performed to reach these selected variables.

### Data processing and analysis

For data analysis, the Stata software, version 16.0, was used. The vaccination goals for D2 of the HPV vaccine were estimates by year and by SRS and GRS.

For trend analysis, the Prais-Winsten autoregressive model was employed, using the vaccination rate (by year and by SRS and GRS) as dependent variables and the year of the study as independent variable (2016-2020).

For this, the rate of vaccine coverage *per* year, per SRS and GRS had to be transformed to a logarithmic scale to reduce the heterogeneity of variance of the residues from the time series regression analysis.

After the analytical procedure of trend analysis, the annual percent change (APC) was performed for the dependent variable analyzed. The following formula was used: APC=(-1+10 [b1]*100%), in which b1 refers to the angular coefficient of Prais-Winsten regression. For the entire analytical procedure, the significance level of 5% was adopted. For the calculation of the 95% confidence interval (95%CI) of the PCA measurements, the following formulas were used: 95%CI minimum=(-1+10 [b1-t*e]*100%); and 95% CI maximum=(-1+10 [b1+t*e ]*100%), in which the values of coefficient b1 (standard error) were generated by the statistical analysis program; the t refers to the 95% percentile of the T-student distribution test and corresponds to 4 degrees of freedom (2.776), which refers to the five years of analysis.

Panel analysis was also performed, since this type of analysis makes it possible to identify structural changes in the relationship between the dependent and independent variable, considering that it accompanies a given sample of individuals (regions) in time. In total, 28 SRS/GRS from the state of Minas Gerais were evaluated, all of which presented complete data in all variables, thus constituting a balanced panel analysis by SRS/GRS and by year of analysis. The random effect model was adopted, since regional variations are identified by random oscillations around a constant mean value, thus being more efficient and with less variability. Panel regression is demonstrated by the formula below:

In the formula, Y represents the dependent variable, *β* represents the variation observed in Y when the independent variable, X, increases in one unit and *ε* indicates the stochastic nature of the model. Subscript i indicates that observations are indexed by case. Subscripts i and t report that the observations are indexed, respectively, by GRS and time. The model becomes more powerful since information is accumulated on the relationship between sociodemographic and socio-environmental conditions for all GRS and years.

Finally, the Hausman test was performed to verify the consistency of the model.

### Ethical aspects

Since this study used free access data, the project was not required to be submitted to the Research Ethics Committee, according to resolution 466/2012 of the National Health Council^17^.

## Results

This analysis resulted in 140 annual observations regarding HPV vaccination rates in the period from 2016 to 2020, related to the 19 SRS and 8 GRS in the State of Minas Gerais, Brazil.

Table 1 shows HPV vaccine coverage rates by age group, year, and trend. We observed that the variations in coverage rates had a higher proportion in the 9-year-old age group, and in 2017 the highest rate occurred for D1. On the other hand, the lowest rate occurred for the older ages, with the 13-year-old age group being the one that stood out the most from the other groups. Regarding trend analyses, we found that only the 9-year-old age group was increasing, and all other age groups were decreasing.

**Table 1:** Analysis of HPV vaccine coverage in the State of Minas Gerais. Belo Horizonte, State of Minas Gerais, Brazil, 2016-2020

Age group (years)	2016		2017		2018		2019		2020		APC* (95%CI^†^)		Trend	
	Coverage		Coverage		Coverage		Coverage		Coverage					
	D1^‡^	D2^§^	D1^‡^	D2^§^	D1^‡^	D2^§^	D1^‡^	D2^§^	D1^‡^	D2^§^	D1^‡^		D2^§^	
9	53.47	21.14	60.82	31.49	57.5	30.15	55.08	27.64	52.28	25.69	-1.29 (-3.79; 1.28)^|^	Increasing	4.11 (-0.02; 8.41)	Increasing
10	9.75	17.41	13.65	27.6	8.31	20.58	8.77	19.79	8.01	18.47	-8.82 (-12.70; -4.77)	Decreasing	-3.83 (-7.08; -0.46)	Decreasing
11	3.42	11.51	5.58	9.17	3.83	8.19	3.48	7.21	4.1	8.18	-22.45 (-28.60; -15.76)	Decreasing	-20.84 (-24.84; 16.62)	Decreasing
12	2.63	9	5.41	6.99	1.78	3.92	1.38	3.42	1.57	3.66	-22.45 (-28.60; -15.76)	Decreasing	-20.84 (-24.84; -16.62)	Decreasing
13	1.25	3.43	4.2	6.38	1.21	2.62	0.77	1.68	0.83	1.83	-24.21 (-70.25; 93.07)	Decreasing	-23.43 (-28.20; -18.34)	Decreasing
Total	13.33	12.22	17.12	15.87	13.73	12.63	13.13	11.51	12.63	11.16				

*APC = *Annual Percent Change;* ^*†*^ CI = Confidence Interval; ^‡^D1 = First dose; ^§^D2 = Second dose

Table 2 shows the coverage rate of the HPV vaccine related to D2 by SRS/GRS *per* year. We observed that all regions presented rates lower than expected for D2 coverage; these rates show a stationary trend over the years. SRS Alfenas exhibited the highest D2 rate in 2017 (42,16) and the SRS of Patos de Minas had the lowest rate, in 2016 (11,2).

**Table 2:** D2 coverage rate of HPV vaccine in the 9-year-old age group, by SRS/GRS, in the State of Minas Gerais. Belo Horizonte, MG, Brazil, 2016-2020

Regional	D2* vaccination coverage (9 years)					PCA^†^ ( 95%CI^‡^)	Trend
	Year						
	2016	2017	2018	2019	2020		
SRS^§^ Alfenas	32.83	42.16	38.2	34.51	33.19	1.19 (-1.38; 3.83)	Stationary
SRS^§^ Barbacena	27.94	35.87	36.52	31.83	29.86	1.57 (0.56; 2.60)	Stationary
SRS^§^ Belo Horizonte	22.09	31.18	30.82	28.11	25.98	0.98 (-1.31; 3.31)	Stationary
SRS^§^ Coronel Fabriciano	20.35	27.35	28.49	30.12	26.9	0.89 (-5.86; 8.12)	Stationary
SRS^§^ Diamantina	17.62	26.68	21.88	24.89	23.95	0.67 (-1.59; 0.26)	Stationary
SRS^§^ Divinópolis	20.51	33.91	30.73	31.56	30.04	3.53 (0.08; 7.10)	Stationary
SRS^§^ Governador Valadares	18.75	27.26	26.34	24.03	25.61	0.26 (-3.66; 4.35)	Stationary
GSR ^||^ Itabira	27.43	37.56	37.91	35.53	29.86	1.25 (-5.32; 8.26)	Stationary
GRS^||^ Ituiutaba	20	36.39	28.27	29.8	26.35	1.26 (-1.27; 3.86)	Stationary
GRS Januária	20.15	28.23	29.84	24.31	22.94	3.20 (-0.07; 6.58)	Stationary
SRS^§^ Juiz de Fora	21.44	27.94	23.1	14.07	15.98	1.21 (-2.21; 4.74)	Stationary
GRS^||^ Leopoldina	16.41	26.25	28.05	29.54	24.58	3.08 (-1.22; 7.57)	Stationary
SRS^§^ Manhumirim	17.82	27.49	28.63	30.21	23.11	4.20 (-0.99; 9.66)	Stationary
SRS^§^ Montes Claros	21.53	31.24	25.23	23.39	23.25	6.40 (-23.80; 14.98)	Stationary
SRS^§^ Passos	19.76	31.82	27.08	24.05	22.68	0.80 (-1.09; -0.51)	**Decreasing**
SRS^§^ Patos de Minas	11.2	29.78	28.42	24.1	25.82	3.11 (-0.29; 6.62)	Stationary
GRS^||^ Pedra Azul	16.77	26.16	24.9	23.09	19.8	4.51 (-0.95; 10.27)	Stationary
GRS^||^ Pirapora	15.85	23.22	19.81	20.92	20.6	6.19 (-2.53; 15.69)	Stationary
SRS^§^ Ponte Nova	21.17	37.68	36.17	29.77	29.45	6.87 (4.51; 9.28)	**Increasing**
SRS^§^ Pouso Alegre	22.81	33.82	32.59	31.01	27.87	2.29 (-0.06; 4.70)	Stationary
GRS^||^ São João Del Rei	26.54	36.46	37.45	34.48	27.25	6.18 (-1.02; 13.91)	Stationary
SRS^§^ Sete Lagoas	21.27	37.75	32.04	28.69	27.63	3.80 (-3.63; 11.81)	Stationary
SRS^§^ Teófilo Otoni	20.35	29.12	30.15	24.07	25.58	1.78 (-1.14; 4.79)	Stationary
GRS^||^ Ubá	18.99	32.43	29.16	29.45	28.36	2.92 (1.31; 4.56)	**Increasing**
SRS^§^ Uberaba	19.91	33.05	28.99	30.17	24.74	1.16 (-2.38; 4.83)	Stationary
SRS^§^ Uberlândia	19.3	31.59	40	29.19	23.44	1.26 (-3.08; 0.59)	Stationary
GRS^||^ Unaí	16.57	27.65	26.09	21.33	18.35	6.25 (-1.01; 14.05)	Stationary
SRS^§^ Varginha	22.73	36.75	32.86	30.93	28.32	2.22 (-0.12; 4.63)	Stationary

*D2 = Second dose; ^†^APC = *Annual Percent Change;* ^*‡*^ CI = Confidence Interval; ^§^SRS = Regional Health Superintendence; ^||^SRG = Regional Health Management

The regions of Ponte Nova and Ubá have shown an increasing trend over the years. In Passos, the opposite (descending) occurred.

Table 3 presents the data regarding the environment over the years. For the variables related to the human development index (HDI), data were presented only for the year 2016, since this variable did not change over the years, since its estimate was carried out by the 2010 demographic census.

**Table 3:** Environmental data of the State of Minas Gerais. Belo Horizonte, State of Minas Gerais, Brazil, 2016-2020

Environmental Variables	2016	2017	2018	2019	2020
	Median (IQ*)	Median (IQ*)	Median (IQ*)	Median (IQ*)	Median (IQ*)
MHDI^†^	0.674 (0.643-0.700)				
MHDI^†^ income	0.661 (0.635-0.693)				
MHDI^†^ longevity	0.825 (0.8136-0.8430)				
MHDI^†^ education	0.570 (0.539-0.591)				
Violence Rate	375.7 (218.66-624.64)	311.105 (240.35-535.91)	242.77 (172.905-341.34)	176.585 (130.605-266.13)	127.08 (103.395-189.57)

*IQ = Interquartile interval; ^†^MHDI = Municipal human development index

Finally, the panel analysis (Table 4) shows the final model of the study, balanced by SRS/SRG and by year of analysis, for the group of 9-year-olds, due to its power of significance.

**Table 4:** Final model for the group of 9-year-old in the State of Minas Gerais. Belo Horizonte, State of Minas Gerais, Brazil, 2016-2020

Coverage rate - D2*			
Variables	Coefficient B	Standard error	p-value
Coverage rate D1^†^	0.5795297	0.497363	0.000
MHDI^‡^ longevity	-588.4987	645.1112	0.364
Violence Rate	-0.0082914	0.0019624	0.000

*D2 = Second dose; ^†^D1 = First dose; ^‡^MHDI = Municipal human development index; Model explanation rate: 93.2%

The independent variables coverage rate of D1, HDI longevity and rate of violence significantly interfered in the coverage rates of D2, presenting with a p-value<0.005; this model was explained with a power of 93.2% of statistical significance.

Regarding the consistency of the model, the p-value was 0.0025 by the Hausman test, which justifies the use of the fixed effects model being more significant for this study.

## Discussion

The results of this study showed that HPV vaccine coverage rates in the state of Minas Gerais are well below the expected targets for all the SRS/SRG and age groups, and most regions showed a stationary and decreasing trend. Regarding the contextual factors associated with the coverage rate of the HPV vaccine, we observed that the variables of D1 coverage rate, MHDI longevity, and rate of violence influenced the D2 coverage rates.

Among the years of the study, 2017 had a small difference in the coverage rates of D2 compared to the previous year, this result is justified by the fact that 2017 was the year in which the HPV vaccine was introduced for the male adolescent population in Brazil. However, these rates did not reach the expected increasing values, nor the ones obtained in other countries^18^.

Australia, a pioneer country in the introduction of HPV vaccine in its national vaccination program for both girls and boys, currently has good results in reducing the incidence of cervical cancer thanks to the vaccination coverage rate, which ranges from 70% to 80% throughout its territory^18^ ^-^ ^19^.

Brazil was the first country in South America and the seventh in the world to offer the HPV vaccine to boys in national immunization programs. The availability of immunobiological strains for boys also provides cross-protection for girls, in addition to protecting against cancers in the penis, throat, and anus, all diseases that are directly related to HPV^1^ ^,^ ^5^ ^,^ ^18^. However, for this to happen, the coverage rates for this public must be within the established goals^5^ ^-^ ^6^ ^,^ ^18^.

Another finding of this study refers to the fact that the year 2020 presented the lowest coverage rates (0.83 for D1, and 1.83 for D2). This fact is very relevant and worrisome, since it probably relates to the year of the beginning of the coronavirus pandemic (COVID-19).

Studies show that the COVID-19 pandemic had a significant influence on public health and that HPV vaccine coverage was also affected^20^. However, this reality is not exclusive to this vaccine, but it is one shared with other regular vaccines^20^ ^-^ ^23^.

In the comparison between the cohorts, the coverage achieved with the first and second dose indicated that the younger cohorts were more likely to be vaccinated, both by SRS/SRG and per year, presenting an increasing trend. This is an extremely relevant factor within the context of HPV vaccination; the population in this age group is the one that benefits most from this immunobiological, since people have not yet been exposed to the viral subtypes of HPV - which makes the vaccine have a greater power of efficacy and immunogenicity against the subtypes of the applied vaccine^8^ ^,^ ^18^ ^,^ ^23^.

This targeted age group of the HPV vaccine is an important and relevant factor for public health, since historically most immunization programs have focused on childhood vaccination. Thu, health services may be less experienced in reaching and vaccinating adolescents^3^ ^,^ ^7^ ^-^ ^8^. This fact can be proven by the low rates of vaccination coverage found in this study, as well as in other countries, since many have limited experience in providing and continuing health care to adolescents, in addition to beliefs/barriers imposed in the context of this immunobiological^6^ ^-^ ^8^ ^,^ ^10^.

In this study, we also found similarity in vaccination coverage rates among the regions of Minas Gerais (SRS/GRS), which may be associated with regional and even state-level public policies.

To improve HPV vaccine coverage rates, low- and middle-income countries, such as India, Peru, Uganda, and Vietnam, have adopted vaccination strategies within community and schools. They emphasize that, to increase the acceptability of this vaccine, other interventions are needed, such as sex education, knowledge, and good health practices. Many of these actions are performed by the nursing professionals^8^ ^,^ ^18^ ^-^ ^19^ ^,^ ^22^ ^-^ ^23^.

Regarding the characteristics of the social environment, the analysis presented here suggests an association between low vaccination coverage and high crime rates by region. Studies show that regions with higher value of this indicator can represent both more rural areas and urban areas of low economic level, which causes strong consequences for economic, social, and health development^23^ ^-^ ^25^.

Geographical barriers can also influence the reduction of access to vaccination services^24^. The SAGE group related, for example, geographical barriers to the hesitation to vaccinate. This occurred when the population was motivated to receive a vaccine but hesitated due to the fact that the health center was distant, or its access was hindered^9^ - this difficulty may be imposed by the insecurity of going to the health service^24^ due to the rates of violence in each region.

Thus, crime can directly interfere in the performance of health care by limiting access to services, causing the population not to attend the health unit^25^ ^-^ ^27^. Again, we emphasize that, to our knowledge, there are few national studies that address the impact of crime index, especially on the health of adolescents.

Territorial characteristics, such as regional inequalities and the peculiar characteristics of each state/region (public security policies, economic infrastructure, health, education, and demographic structure), are extremely important factors for the development of public health strategies^18^ ^-^ ^19^ ^,^ ^23^. When developing strategies to raise awareness of the importance of HPV vaccination, it is necessary to adapt them according to the socioeconomic and socio-environmental characteristics of the location. In this case, the nursing professional is able to contribute to this process through situational analysis of the population and with the use of tools, such as situational diagnosis^7^ ^,^ ^27^.

Moreover, nurses within health centers develop actions to prevent diseases and promote health, which includes one of the measures of early diagnosis of cervical cancer - the performance of the Pap smear test. Thus, nurses are able, for example, to analyze the rates of altered tests, as well as the coverage rates of the HPV vaccine, and to develop strategies that encourage adolescents to seek vaccination. The strategies adopted for the adolescent public and the HPV vaccine are very recent in our country, mainly due to the taboo surrounding this vaccine^7^ ^,^ ^27^.

Finally, this study presents some limitations: the research was developed based on data from secondary databases, which presented limited information (such as the data not being segregated by gender), in addition to the model being adjusted only for the 9-year-old age group, due to the fact that the other age groups have very low vaccination coverage rates.

Despite the potential limitations, we emphasize that the goal of HPV vaccine coverage by the year 2030 is aligned with the coverage targets of 90% to 95% established for other childhood vaccines, such as diphtheria-tetanus-pertussis (DTaP) and measles vaccines^21^. Despite all the challenges, including those related to the social environment (such as crime rates), for the state of MG to become a reference in relation to the vaccination of adolescents and to reach the recommended targets for the HPV vaccine, it is necessary to intensify of public policies aimed at health education, as well as the awareness of the target audience of the vaccine and of managers and health professionals - especially nurses - about this very important issue. The professional nurse undoubtedly has a fundamental role in the implementation of such actions and vaccination strategies for adolescents, considering all their care specificities. They play a fundamental role in the implementation of such vaccination actions and strategies for adolescents, considering all their specificities of care.

## Conclusion

The evidence found in this study may contribute to improve the understanding of the complex relationship between environmental and individual determinants and vaccination, which may play an important role in expanding public health strategies and policies. We aim to contribute to the increase in vaccination coverage rates in adolescent public not only within the State of Minas Gerais but also within the entire country.

The analysis of panel data, social environment, and vaccination is recent in the field of public health, but it is extremely important. This study confirms that, although tenuous, aspects of the environment, in addition to individual characteristics, provide relevant information for the understanding of the occurrence of health outcomes: the impact of HPV vaccine rates in terms of public health will occur if 90% of vaccination coverage is reached among adolescents. The effective operationalization toward this goal is extremely relevant and necessary for the eradication of cervical cancer and other HPV-associated diseases/neoplasms.
